# Small Pox Appearing in a Family without Any Previous Chance of Contagion

**Published:** 1849-09

**Authors:** E. C. Banks

**Affiliations:** Lawrenceville, Ill.


*Small Pox appearing in a family without any previous chance of Contagion.* By E. C. Banks, M. D., of Lawrenceville, Ill.

Mrs. G——, aged about 20 years, of robust constitution, pregnant 5 months, was attacked by a chill followed by fever of a moderate grade, remitting every morning, which continued for seven days. At the end of the seventh she commenced being chilly, which lasted for twenty-four hours; then her fever again arose and continued three days, when an eruption of a minute red papulæ appeared on her face, head and arms, and coalescing at their base. In a few hours more they could be seen making their appearance all over her body. In twenty-four hours they were well out, and fast running together. At this time they assumed a vesicular form. On the third day after the eruption first appeared, it was still more prominent, and the vesicles filled with coagulable lymph, of a dull whitish color; and surrounding each vesicle was a distinct areola. Fourth-day. Eruption still increasing, and becoming more prominent, and more yellowish than before. Fifth-day. Integuments much swollen, fever increased considerably, with an immense flow of saliva, pustules more turgid, and of a spherical form, flattened on the top; local inflammation and pain increased. Sixth-day. Much the same condition as on the fifth, swelling rather greater, fever quite as high; pus could also be seen in each pustule on the face, head and arms. All the symptoms now seemed more aggravated. Seventh-day. Condition much the same as on the sixth, fever rather less, thirst not so great. Eighth-day. Pustules still more flattened, and pitted in the centre, swelling of the integuments abated. They were now at their full size, and were completely confluent. The eyes nearly closed from the swelling around them, and in the eyelids. The eruption was worst on her face. Ninth day. A few pustules on her face had broken, and thin incrustations formed. The tenth day still more had burst, and discharged a thin ichorous fluid, which formed thin, soft incrustations of a brown color. The twelfth day her face seemed one entire brown scab, with here and there a portion peeling off.

Fourteenth day. Still desquamating on her face, head and arms, while on her body it was commencing to do so; though the eruption came out later on her body and legs, it began to dry rather earlier than it did on her face and head. Fifteenth day. Desquamation in full blast. Great itching now present. Secondary fever now arose, and before she had scaled off, she sunk and died, not however without having aborted about the tenth day of the eruption. The attending physicians did not consider it a case of small pox; but as others had taken it about this time, I was sent for, and found a near neighbor of this family laboring under variola. I went immediately to see the dead body, which I found well marked with the same disease. I at once sounded the alarm, by telling them it was small pox.

The family in which the first case occurred, lived completely in an out of the way place; no one ever visited them but their neighbors, and no small pox had been within a hundred miles of that place, nor had any communication taken place between them and any strangers, either pedlars or others. No clothing had been purchased except a shawl at one of the stores in our village, dozens of which out of the same pack had been sold to other persons, and no ill consequences had followed. They are people that keep at home most completely, and as I stated above, no communication ever takes place between them and strangers. Hence the impossibility of its being brought there, the man of the house positively asserting that no intercourse has occurred, and that no garment, except the said shawl, has been purchased for six months. All those who had been vaccinated escaped altogether, or with varioloid in the mildest form. Several very severe cases occurred among those who never had been vaccinated.

A consulting physician who was sent for from a neighboring city, Vincennes, Ia, decided it to be genuine small pox. It has spread greatly in consequence of the assurance by others than myself that it was not contagious. The eruption was confluent in a few, but distinct in the majority. In those who have had it in the confluent form, dangerous symptoms have arisen, and three have died who had it in that form, while four recovered. All the others got well who had it in the distinct form. I omitted to mention that the fœtus which this lady lost was completely covered with the eruption; it only lived three hours.

I am of the opinion that small pox occasionally originates without the influence of contagion, and that this was one of the cases of that kind. I see no possibility of her having taken it from any clothing purchased, not even from the shawl; and if so, others certainly would have taken it in the same way, as the shawls were sold to different persons out of the same pack. The fact, also, that she was sick seven days before the particular symptoms of small pox made their appearance, while in all the other cases who have taken it from her, and others who have taken it from them again, the eruption commenced making its appearance about the latter part of the third or beginning of the fourth day, militates against the idea of contagion.

[In connection with the above it may be stated that two cases have recently occurred in the Eastern Penitentiary of Pennsylvania, in which the " separate system" is strictly enforced. In one case the convict had been incarcerated for two years, and in the other for six. In these instances it would be difficult to explain the occurrence of the disease by contagion.—*Editors Examiner.*]

